# A Critical Review of the Design, Manufacture, and Evaluation of Bone Joint Replacements for Bone Repair

**DOI:** 10.3390/ma15010153

**Published:** 2021-12-26

**Authors:** Yi Huo, Yongtao Lyu, Sergei Bosiakov, Feng Han

**Affiliations:** 1 Department of Engineering Mechanics, Dalian University of Technology, Dalian 116024, China; 201790215@mail.dlut.edu.cn; 2 DUT-BSU Joint Institute, Dalian University of Technology, Dalian 116024, China; 3 Faculty of Mechanics and Mathematics, Belarusian State University, 220030 Minsk, Belarus; bosiakov@bsu.by; 4 Department of Hand Microsurgery, The First Affiliated Hospital, Dalian Medical University, Dalian 116011, China

**Keywords:** orthopedic implant, bionic design, additive manufacturing, numerical evaluation

## Abstract

With the change of people’s living habits, bone trauma has become a common clinical disease. A large number of bone joint replacements is performed every year around the world. Bone joint replacement is a major approach for restoring the functionalities of human joints caused by bone traumas or some chronic bone diseases. However, the current bone joint replacement products still cannot meet the increasing demands and there is still room to increase the performance of the current products. The structural design of the implant is crucial because the performance of the implant relies heavily on its geometry and microarchitecture. Bionic design learning from the natural structure is widely used. With the progress of technology, machine learning can be used to optimize the structure of bone implants, which may become the focus of research in the future. In addition, the optimization of the microstructure of bone implants also has an important impact on its performance. The widely used design algorithm for the optimization of bone joint replacements is reviewed in the present study. Regarding the manufacturing of the implant, the emerging additive manufacturing technique provides more room for the design of complex microstructures. The additive manufacturing technique has enabled the production of bone joint replacements with more complex internal structures, which makes the design process more convenient. Numerical modeling plays an important role in the evaluation of the performance of an implant. For example, theoretical and numerical analysis can be carried out by establishing a musculoskeletal model to prepare for the practical use of bone implants. Besides, the in vitro and in vivo testing can provide mechanical properties of bone implants that are more in line with the implant recipient’s situation. In the present study, the progress of the design, manufacture, and evaluation of the orthopedic implant, especially the joint replacement, is critically reviewed.

## 1. Introduction

Bone trauma is a serious disease affecting the whole population worldwide. In many instances of bone trauma, especially those occurring in elderly people, bone joint replacement surgery has to be performed. Among these surgeries, hip and knee joint replacements are very common. According to statistical data, more than one million total hip replacements are performed each year in the world. In 2017, approximately 37,000 primary total hip replacements were performed in Australia and 97,000 in the UK [1]. Due to an increase in outdoor and sports activities combined with unhealthy food, the hip joints can thus be damaged. Nowadays, people’s eating habits are very bad for bone health. For example, an excessive intake of salt will cause the loss of calcium, which in turn affects the health of bones. Patients suffering from joint pain and undergoing total joint arthroplasties, including total hip arthroplasties (THA), are getting younger and younger [2]. Due to these high demands, many different bone joint replacement products have been designed and relevant worldwide companies have become well established, e.g., DePuy, Johnson & Johnson, Smith & Nephew. The installation of joint replacements has enabled the restoration of human joint movement and, therefore, the daily activities of the human. However, there are still many issues related to the current joint replacements. For example, the wear and the micro-motion at the interface of the joint are still unsolved challenges, which cause the early failure and loosening of the joint replacement products [3]. The dislocation of the femur head is another common issue related to joint replacement. The design of the shape and microstructure of the joint replacements is one effective approach to solve these challenges because the shape and microstructure of the replacements can alter the load distribution and consequently the issues surrounding wear and head dislocation might be relieved [4].

In the field of bone joint replacement, as design approaches and manufacturing techniques advance, bone joint replacements have changed significantly and their performance has improved significantly. Design, manufacture, and evaluation are three important aspects of bone joint replacement. They are interconnected and influence each other. For example, advanced manufacturing technology enables the design of joint replacements with complex internal structures. Performance evaluations of joint replacements push the development of manufacturing. Therefore, in the present study, a critical review of the design, manufacture, and performance evaluation of bone joint replacements is provided to advance further developments in this field.

## 2. Review of the Design of the Bone Joint Replacement

**Figure 1:**
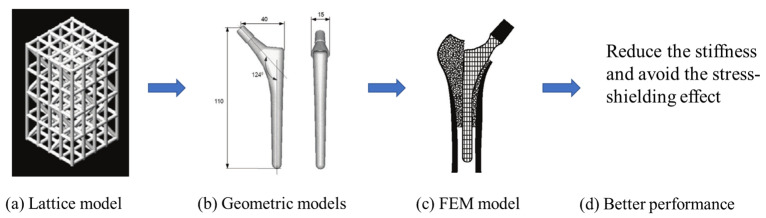
An example of obtaining better performance by designing a new structure. Adapted from reference [6].

The design of a bone joint replacement plays an important role in improving the performance of the joint replacement. The design objectives, the design variables, and the design constraints are the three key elements in the design. Ideally, the bone implant should have similar hierarchical configurations on multiple scales. Besides, the implant should possess properties similar to the host bone to match the mechanical performance. Therefore, the implant should possess both adequate stiffness to resist the physical loading and sufficient permeability since the transportation of cells requires the flow of blood through the implants [5]. Regarding the design objective for the design of bone joint replacements, hip and knee implants are the main objects of study due to their high level of demand. Regarding the design variables for the design of bone joint replacements, several studies have analyzed the size and profile of the implant. On the other hand, the stiffness of the implants is also very important, because the stiffness is closely related to the stress-shielding effect. A high stem stiffness means that most of the load is transferred from the prosthetic head to the distal femur by the stem itself. So, the bone tissue load in the epiphyseal region of the proximal femur is significantly lower than the physiological load. This effect is called ‘stress shielding’. Therefore, a structure with proper stiffness should be selected as the implant to prevent the stress-shielding effect caused by high stiffness [6]. Ścigała et al. [6] used internal lattice structures to reduce the stiffness of the hip endoprosthesis. They designed new structures and used the finite element method to analyze the stiffness of the implants. Their results showed that the use of inner lattice structures reduced implant stiffness and therefore potentially avoided the stress-shielding effect (Figure 1).

Design constraints for the design of bone joint replacements mainly occur due to technical limitations or design precision requirements. For example, the porous microstructures that have emerged in recent years can be manufactured by additive manufacturing (AM), but the accuracy of printing should be improved to meet the requirements. As mentioned above, patients are getting younger and younger, and thus Shaik et al. [7] investigated the durability of the implant and the effects of the size of the femoral ball on the stress state in the implant were investigated. The durability of the implant is determined by the bio-compatibility of materials, wear characteristics, and the implant shape [7]. The durability of the implant can fully reflect its working life compared with fatigue (the initiation and propagation of cracks in a material due to cyclic loading). In Table 1, the 2-D model established by the author is shown. The main research objects are D region (neck region) and E region (contact region) in the figure, the regions A, B and C are fixed. Kladovasilakis et al. [8] minimized the stiffness of the prosthesis to avoid stress shielding and minimize the implant’s weight at the same time. Minimizing the weight is defined as maintaining the desired mechanical properties while reducing the mass of the structure. Abdellah et al. [9] introduced a novel methodology to realistically design cemented hip prostheses by controlling the size of the implant cross sections, and they minimized Young’s modulus in this way. Thus, the stiffness is minimized to avoid stress shielding. However, further in vivo experiments need to be conducted to verify this conclusion. As mentioned above, the stiffness of the structure has an important influence on its mechanical and biological properties. Thus, the stiffest design method is an effective optimization method. The stiffest design method generally comprises the optimization of size, shape, and topology. Nowak et al. [10,11] have conducted a lot of research on the stiffest design through mathematical models. In their work, the main assumption is that there is a constant strain energy density on the structural surface. The compliance, C, is defined as the work of the given loading performed on the displacements caused by the same loading [10]. The compliance can be defined by mathematics as [10]:

(1) $$J\left(\Omega \right)=\int_{\Gamma_{1}}\;t\cdot u\;ds$$

The goal of the stiffest design method is to maximize the stiffness of a structure. In this case, the stiffness of the structure is equal to the inverse of the compliance, which means that the goal of the stiffest design method is to minimize the compliance, C. Thus, the goal is to minimize Equation (1). Regarding the constraint in this case, the volume of the material in the design domain is usually limited [11]. The constraint is the given volume which defined as [10]:

(2) $$\int_{\Omega_{0}}\;dx-V_{0}=0$$

For the standard elasticity system, the state equations are defined as [10]:

(3) div σ(u)=0  in Ω

(4) $$\sigma \left(u\right)\cdot n=t\;\;\;\;\mathrm{on}\;\Gamma_{1}$$

(5) $$\sigma \left(u\right)\cdot n=t\;\;\;\mathrm{on}\;\Gamma_{v}$$

(6) $$u=0\;\;\;\;\;\;\;\;\;\;\;\;\;\mathrm{on}\;\Gamma_{0}$$

where Ω represents the domain of the elasticity system, u represents the displacement, σ(u) represents the stress tensor, $\Gamma_{0}$ represents part of the boundary with the Dirichlet condition, $\Gamma_{1}$ represents part of the boundary loaded by traction forces t, and $\Gamma_{v}$ represents part of the boundary subject to modification.

Regarding the optimization of size of the implants, Nowak et al. [10] established the enhancement of the trabecular bone remodeling regulatory model, based directly on the optimization of shape. The results showed that the equalization of the strain energy density on the trabecular bone surface minimizes the strain energy on the whole structure of the bone, which proved that the remodeling process which leads to the formation of the structure with the highest stiffness is the correct model to use.

Regarding the optimization of topology, Nowak et al. [11] eliminated the volume constraint from the topology optimization procedure, based on the trabecular bone remodeling phenomenon, which means the Lagrange multiplier is assumed to have a constant value. Based on that assumption, compliance was minimized by obtaining different topologies for different materials. Additionally, it is also possible to obtain different topologies for different load magnitudes based on this assumption.

Several representative optimization methods focusing on hip implants are summarized in Table 1.

As design methods have advanced, both the exterior shape and the internal microstructures of bone joint replacements have evolved. In the early days, typical designs for artificial hip replacements were based on a ball and socket with three different choices of material combination acting as the bearing surfaces [12]. From the microscopic point of view, the traditional structures are dense and stiff. Therefore, the traditional structures produce a stress-shielding effect since they modify the original load-sharing path in the bony structure, which leads to bone resorption and implant loosening. Nowadays, the introduction of porous microstructures has improved the mechanical properties of implants. For example, lightweight structures have been achieved and stress shielding has been reduced (Table 2). In addition, the functionally graded implants can better meet all kinds of practical requirements. For example, the functionally graded implant can change the structural stiffness in a gradient to adapt to the actual bone growth environment. As mentioned above, the introduction of porous microstructures and functionally graded materials (FGMs) are two methods of realizing the optimization of mechanical properties.

In recent years, internal porous microstructures are used in joint replacements, so the microstructures of the joint replacement need to be designed. It should be noted that human bones and joints, including jaws and femurs, are not completely solid. The interconnected porous structure not only facilitates the inflow of nutrients and the export of metabolic waste but also provides good conditions for cell growth and attachment. Additionally, stress shielding can be avoided to a certain extent when porous structures are used instead of solid structures [13]. It is worth noting that porous structures have a variety of design methods, and that a cellular structure is a kind of porous structure designed in the early stage, such as honeycombs. Cellular structure can be observed in the bones of birds and in shells, this kind of structure possesses strength and low weight [14]. In recent years, porous structures based on triply periodic minimal surfaces (TPMS) have attracted the attention of researchers due to their strong mechanical performance, such as high surface area–volume ratio, full connectivity, high smoothness, and controllability [15]. Kolken et al. [16] assessed the mechanical properties of additively manufactured architected materials made from cellular structure unit cells; the representative example in Table 2 shows the cellular structure. Bruno et al. [17] produced two femoral stems: the first was fully dense, while the second featured a diamond (TPMS) structure in its core. The results obtained predicted less bone resorption in the femur implanted with the porous stem than the bone implanted with its dense counterpart. In the future, a unified multilevel design framework should be established to design the exterior shape and microstructure of the joint replacement at the same time.

FGMs refer to the introduction of the spatial variation gradient of composition and/or microstructure into the material so that the performance of the material changes in a gradient fashion in the spatial position. Through this kind of design, the local stress concentration can be reduced. At the same time, different parts of the structure can complete their functional tasks under different working conditions and the integrity and reliability of the whole structure can be ensured [18]. Several studies have demonstrated the suitability of FGMs for use in various prostheses including hip, knee, and dental implants [19]. Abdellah et al. [9] designed functionally graded implants to optimize the mechanical properties of the implants. The functionally graded implant was sliced into eleven lengthwise layers, with each layer possessing a different Young’s modulus. As a result, stress shielding may reduce if the proper Young’s modulus (need further in vivo tests) and interface shear stress were also reduced. The above method is used to introduce materials with gradient changes; another method is to design the microstructure of a functional gradient. For example, Kladovasilakis et al. [8] manufactured functionally graded lattice structures to achieve better mechanical performance; the mechanical bearing capacity of the new functionally graded implant was twice as much as that of the normal implant in vivo experiments. The evolution of the hip joint replacements is summarized in Table 2.

**Table 2:** The evolution of hip joint replacements.

Development Stage	Representative Structure		Representative Example	References
Early stage (approximately before 2002)	Typical structure		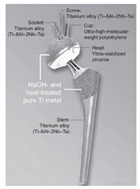	[12]
Nowadays (approximately 2002 to present)	Porous materials	Internal cellular structure	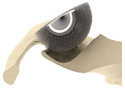	[16]
		Internal TPMS structure	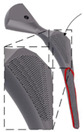	[17]
	Functionally graded implant	Functionally graded material	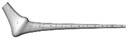	[9]
		Functionally graded microstructure	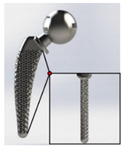	[8]

Table 2 summaries the evolution of the microstructures of implants; porous microstructures and functionally graded materials are widely used in the design of the implant. Additionally, the metamaterials have drawn the attention of many researchers, since some special properties can be achieved using metamaterials, such as the super-toughness, invisibility, etc. [20]. Metamaterials can be divided into auxetic and non-auxetic structures, the difference is that auxetic structures have the negative Poisson’s ratios. Usually, when we stretch a material, the material not only becomes longer in the direction of stretch, but also becomes thinner in its cross-section. This behavior is defined by the fundamental mechanical property of materials: the Poisson’s ratio, which represented by ν. The Poisson’s ratio of a material is defined as the ratio of the lateral contractile strain to the longitudinal tensile strain for a material undergoing tension in the longitudinal direction [21]:

(7) $$\nu_{xy}=-\frac{\varepsilon_{y}}{\varepsilon_{x}}$$

where $\varepsilon_{y}$ is the lateral strain generated in response to the axial strain $\varepsilon_{x}$. Consequently, most materials have a positive Poisson’s ratio, where ν > 0. As mentioned above, the auxetic structure exhibits the negative Poisson’s ratio (NPR) effect, which means it can expand transversally when axially expanded under tensional force. There are three main types of auxetic structure: the re-entrant, the chiral, and the rotating structures. Regarding the re-entrant structures, they can be formed by an arrowhead structure, a star shape structure, a missing rib structure, etc. [22]. For the re-entrant structure, Kolken et al. [23] showed that it can restore the bone–implant contact on the lateral side of a hip stem and the fatigue life will be long enough. The structures with deformation dominated by rotational reflection that exhibit the NPR effect are called chiral structures [22]. In contrast to most of other auxetic structures, this structure can maintain a high auxetic effect over a significant range of strains [21]. Yao et al. [24] designed different bone screws based on re-entrant structures, chiral structures, and rotating structures. Then, they investigated the mechanical properties and fixation strengths of each. The results showed that the auxetic bone screws composed of re-entrant structures and chiral structures possessed higher tensile stiffness and strength. Rotating structures have applications in all kinds of fields of life: cars, aero-planes, vacuum cleaners, and steam-turbines all contain many rotating structures [25]. The reliability, stability, and response levels of these machines, predicted by analytical models, are generally not satisfactory until validated by experimentally obtained data. At present, the widely studied standardized model is rigid squares that are connected through simple hinges at their vertices. For an ideal system, which is isotropic when a tensile loading is applied, the squares rotate at the vertices, the whole structure expands, and the Poisson’s ratio ν equals −1. Table 3 summaries the auxetic structures and their applications in bone implants.

Effective design methods must be used to design joint replacements. In the past, design methods have advanced significantly. The design methods can be classified into the following three main types. The first type is the finite element method (FEM), which is a widely used approach to design and analyzes the structure of bone joint replacement. Usually, researchers will first establish mathematical models or computational models, and then use the FEM to verify the accuracy of the models and optimize them. For example, the idea of the mathematical model method is to establish mathematical expressions to control the nature of the implant, such as the geometric properties of the cross section, etc., and then to use the finite element method to investigate the mechanical performance of the implant. Based on the results, the optimization of the mathematical model can be obtained. The second method is the meshless method. This generally uses more nodes to construct the shape functions when compared with the FEM, which makes it a more precise method. In the meshless method, the solid domain is discretized using randomly distributed nodes or collocation points, which are able to approximate the field function within a flexible influence-domain rather than a fixed size element [26]. The two widely used meshfree methods are radial point interpolation and moving least squares [27]. It is worth noting that the cell density of the background mesh and the number of the Gauss points must be precisely balanced in order to obtain good results [28]. The third optimization approach is to use emerging technologies, such as the machine learning technique. These techniques are characterized by complex algorithms that can be trained to reproduce model behavior. The machine learning technique can increase diagnostic accuracy and reduce costs and human resources [29]. Therefore, this technique will become the focus of research in the future.

Cilla et al. [30] reconstructed a femur from CT clinical images and built an FE model. Thus, the strains in the intact and drilled femurs were determined under physiological, patient-specific, muscle, and joint contact forces. Bruno et al. [17] developed a simplified computational model of stress shielding; the finite element method was used to investigate the model. The computational models showed reasonable agreement between the forced placement diagrams obtained by finite element method. In Table 4, the mesh and boundary conditions used by Bruno et al. [17] for the FE analysis are shown as the figure. In the figure, (a) represents the intact femur and (b) represents the implanted femur. Hussin et al. [31] developed a computational model which can be used to assess the wear arising at the implant articulating surfaces. Furthermore, they verified the model using the mechanical test. Belinha et al. [32] used the meshless method to obtain smoother and more accurate strain energy density (SED) fields to predict the bone tissue remodeling. Cilla et al. [33] used machine learning techniques combined with the finite element method to optimize the geometry of a commercial short-stem hip prosthesis. They investigated the effects of different parameters on the performance of the structure. The results showed that decreasing the stem length and reducing the length of the surface in contact with the bone are the two most effective methods to optimize the structure. The widely used design algorithms for the optimization of bone joint replacement, especially the three methods mentioned above are summarized in Table 4.

It should be noted that, in addition to the structural design and optimization, material selection and optimization are also crucial steps in the design of joint replacement. For example, Metal-on-Polyethylene (MoP) bearings, which were popularized by Charnley in the 1970s, are extensively used in THA. It is worth noting that the MoP bearings used in modern hip replacements are better than the MoP bearings used in the 1970s. The specific advantage of MoP bearings is to provide a cost-effective bearing with predictability [34].

## 3. Review of the Manufacturing of the Bone Joint Replacement

The manufacturing of bone joint replacements is closely related to the design of the joint replacements. When designing a joint replacement, the manufacturing processing, constraints, etc., should be taken into account. Otherwise, the designed products may not be producible. Therefore, the design of the joint replacement is largely influenced by the manufacturing techniques. The commonly used manufacturing techniques for producing bone joint replacements can be classified into the following groups: First, traditional manufacturing methods, such as rapid prototyping (RP) and Computer Numerical Control (CNC) [35]. Second, the emerging AM method. The RP approach is widely used in engineering for the quick fabrication of geometrically complex concepts. First, the 3D model of the implants should be constructed and sliced. Then, the computer controls the materials to be deposited on the operating platform to manufacture the bone implants [36]. Regarding the CNC method, the milling strategy and types of tools must be properly selected to achieve maximum accuracy in the resulting physical implant. Marcin et al. [37] presented clinical and technical information on temporomandibular joint replacements, where custom-made implants were manufactured using two different techniques: CNC and direct metal laser sintering (DMLS). In recent years, the emergence and application of the AM technique have enabled the production of bone joint replacements with more complex internal structures.

The AM is an emerging technique, which enables the production of nonhomogeneous and irregular structures. Among the various AM techniques, selective laser sintering (SLS), selective laser melting (SLM), electron beam melting (EBM), and binder jetting (BJ) have been successfully used to produce porous bone implants, such as knee implants. The selection of a repeatable and reliable manufacturing method is essential in manufacturing the design. Several factors can influence the selection of the manufacturing method, including the quantities required, the desired surface finish, the cleaning required post-fabrication, the risk of contamination during manufacturing, packaging, and sterilization [38]. For example, Wauthle et al. [39] manufactured highly porous pure tantalum implants with fully interconnected open pores using the SLM technique. Ataee et al. [40] manufactured Ti-6Al-4V gyroid scaffolds with high porosities using the EBM technique and investigated their mechanical properties. Duan et al. [41] manufactured three-dimensional nanocomposite bone scaffolds using the SLS technique and carried out the compression tests to investigate their mechanical properties. However, there are some constraints that must be considered while using the AM technique. For example, the structure thickness should be no less than 0.2 mm if the SLM is used [42]. Additionally, components with small overhang angles or hanging features may deform when fabricated using laser or electron beams in a layer-wise manner, so the minimal hanging angle should be larger than a set threshold value [43]. The manufacturing techniques used for producing bone joint replacements are summarized in Table 5. The representative examples in Table 5 are the micro-structures of the bone joint replacements observed under a microscope.

## 4. Review of the Evaluation of the Performance of the Bone Joint Replacement

The evaluation of its performance is an important step in the development of a bone joint replacement. An accurate and efficient evaluation of the performance of the joint replacement can be fed back to the design stage so that the design can be improved. However, at the moment, an efficient approach to evaluate the replacement performance is still lacking. The in silico, in vitro, and in vivo testing approaches are the three main types of method used to evaluate the performance of joint replacements.

The in silico method, also called the numerical method, uses the computational models to numerically evaluate the performance of a joint replacement. Once it is developed and validated, it can be efficiently used to evaluate the replacement’s performance. However, at the current stage, the development of valid computational models is still a challenging issue. The numerical models developed in the literature can be classified into three groups. First, the musculoskeletal models are used. The musculoskeletal models are the models using rigid bones, muscles lines, etc., to simulate the activities of the human body. The models can be used to quickly evaluate the influence of the geometry of the replacements on the performance during daily activities, such as stair climbing, squatting, etc. Navacchia et al. [44] proposed a solution to address the inevitable tradeoff between computational cost and model detail in musculoskeletal simulations. However, because the musculoskeletal models are simplified as rigid models, detailed information regarding the stress and strain distribution within cannot be obtained, so the wear mechanism cannot be fully explored. To tackle these issues, the second approach, i.e., the coupled musculoskeletal-FE model, is used. The musculoskeletal-FE model uses rigid segments to represent the human body and uses the FE model to represent the part that is of great interest. The boundary conditions are passed from the musculoskeletal model to the FE model so that the FE model can be used to simulate different stages of daily activities. The advantage of this method is that both the overall and local behaviors in the joint replacement and the human tissues can be evaluated. However, defining the appropriate boundary conditions in the FE model is a challenging issue. The musculoskeletal model is a simplified model, and thus the boundary conditions obtained from it may not be accurate and appropriate. To solve this problem, FE models of the human body have been developed. FE models directly use the FE technique to simulate the daily activities of the human body. The challenge for this technique is to simulate the active contraction of the skeletal muscles, which is the driving force for musculoskeletal movements. The advantage of this approach is that the entire human segments are modeled using the deformable body and, consequently, the detailed mechanical information (stress, strain, etc.) can be obtained in every location within the model. However, because the detailed FE model is built, the computational cost is very large in the simulation of daily activities. Using this technique, Navacchia et al. [44] developed a computationally efficient muscle-force prediction strategy to track gait and chair rise experimental joint motion with a finite element of the musculoskeletal model of the lower limb. Kebbach et al. [45] used a robot-assisted test method based on a musculoskeletal model to investigate a total knee replacement. They investigated many parameters, such as different tibial slopes and different soft tissue conditions. Shu et al. [46] presented a coupled musculoskeletal–FE model to analyze the interactions between prosthetic mechanics and subject dynamics after a total knee replacement (TKR) surgery is performed. Li et al. [47] developed an FE musculoskeletal model including bones, joints, and muscles of the lower extremity to optimize the structure. Shriram et al. [48] evaluated the effects of material stiffness variations on anatomically shaped artificial meniscal implants in the knee joint. Beidokhti et al. [49] investigated the effect of the ligament modeling strategy on the predictive capability of FE models of the human knee joint. The in silico methods for evaluating the performance of knee joint replacements are summarized in Table 6.

The in vitro evaluation method uses lab testing to evaluate the performance of the joint replacement. In general, the following tests are widely used: static mechanical testing, fatigue testing, and wearing testing. Matsoukas et al. [50] developed a computational model to simulate the behavior of in vitro wear, and fatigue testing was carried out. The wear assessments of a total hip replacement (THR) bearing were performed for up to 3 × 10^6^ cycles, and the wear rates for the testing specimens were within the acceptable range. It has been shown in previous studies that the bearing variables, such as the femoral head radius, head-liner clearance, and acetabular liner thickness, are the parameters affecting the volumetric wear [3]. The failure of cemented total joint replacements is often attributed to the loosening of the implant in response to fatigue of the interface between the bone and the cement. Four fresh-frozen human femurs were scanned and loaded in the stance positions at three different angles while recording the strain on the bones’ and prosthesis’ surfaces [51]. Yang et al. [52] characterized the fatigue strength of the bone interface and concluded that fatigue strength increased with the magnitude of the average surface roughness as a result of the increase in interdigitation of cement. Beidokhti et al. [49] optimized the stiffness parameters and pre-strains for a knee joint model based on laxity tests. A fresh-frozen human femur was CT-scanned and thereafter loaded in vitro in a stance position until it fractured at the neck [53]. Based on different testing methods, the widely used in vitro testing methods for evaluating the performance of bone joint replacements are summarized in Table 7.

In vivo testing is the advanced evaluation method. It has a high cost and thus is often used to evaluate the products which have been numerically and in vitro tested. Concerning the in vivo testing, animal testing is normally performed first, followed by human clinical trials. Regarding the clinical trial testing of bone joint replacements, Li et al. [54] implanted the porous scaffold into the goat metatarsus and analyzed the stability of the implantation. Taniguchi et al. [55] manufactured a scaffold using SLM and investigated the effect of pore size on bone ingrowth in rabbits in an in vivo experiment. Ackland et al. [56] designed and implanted a personalized 3D-printed prosthetic joint replacement for the human temporomandibular joint. The implant was designed for a 58-year-old female recipient with end-stage osteoarthritis of the temporomandibular joint. Six months postoperatively, the prosthesis recipient had a normal jaw opening distance (40.0 mm), and no complications were identified. Wegener et al. [57] developed an iron-based degradable sponge-like implant for bone replacement and carried out the in vivo test. It was hypothesized that early implant fixation would be improved by filling the interior of the implant with a carrier providing a spatio-temporal release of bone active drugs with known osteogenic effect. Raina et al. [58] carried out an in vivo test to validate the hypothesis. Du et al. [59] manufactured a bio-inspired multilayered osteochondral scaffold and investigated its cytocompatibility. Warnke et al. [60] repaired an extended mandibular discontinuity defect in the growth of a custom bone transplant inside the latissimus dorsi muscle of an adult male patient. The widely used in vivo testing methods for evaluating the performance of bone joint replacement are summarized in Table 8.

## 5. Conclusions and Future Perspectives

In this study, the design, manufacture, and performance evaluation of the bone joint replacement are critically reviewed. In conclusion, the design of joint replacements has emerged from being design at the single level and single function and has become design at multiple levels and to meet the multiple functions. Regarding manufacturing, the emerging additive manufacturing technique is widely used, which enables the production of joint replacements with complex internal microstructures. Regarding the performance evaluation, the computational model is a crucial approach and will become an efficient method for performance evaluation. However, many developments are still required to move this technique into clinical application. In the future, the development of joint replacements can be performed in the following aspects:

(1) The use of the machine learning in the optimization of the joint replacement.
(2) The use of advanced measurement techniques to validate the computational models and to evaluate the performance of the joint replacement.
(3) The consideration of the long-term dynamic behavior of the joint replacement and the surrounding environment.

## Figures and Tables

**Table 1:** The design of hip joint replacements.

Representative Study	Design Objective	Design Variable	Design Constraints	References
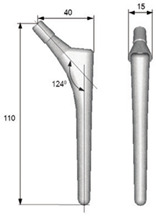	Minimize the stiffness of hip endoprosthesis	The diameter of the internal structure of the stem of the hip joint prosthesis	The thickness of the structure manufactured by AM no less than 0.2 mm due to the manufacturing technique	[6]
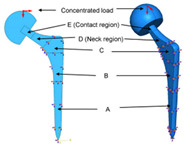	Maximize the durability of the implant	Femoral ball sizes	The natural size of the femoral ball usually ranges from 40 to 54 mm	[7]
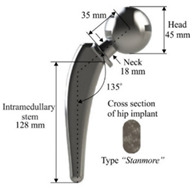	Minimize the weight	The head diameter, the diameter, and length of the neck	The relative density should be 50% due to the femur bone	[8]
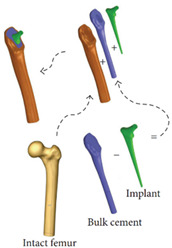	Minimize stress shielding	Shapes and sizes of the cross sections	The accuracy of the computational model needs to be verified	[9]

**Table 3:** The auxetic structures and their applications in bone implants.

Type of Auxetic Structures	Application in Bone Implants	Advantages and Disadvantages	References
Re-entrant	Bone-implant contact; medical screw	Good NPR effect; longer fatigue life	[22,24]
Chiral	Bone scaffold; medical screw	High fracture toughness; limited by chirality	[24]
Rotating	Auxetic materials fabrication; medical screw	Better auxetic performance; low stability	[24]

**Table 4:** The widely used design algorithm for the optimization of bone joint replacements.

Optimization Method		Advantage and Disadvantage	Representative Study	References
Finite element method	Geometrical model	Further in vivo tests are needed	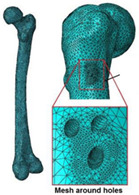	[30]
	Computational model	The model is verified by in vivo testing	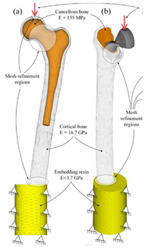	[17]
Meshless method		More accurate; difficult to establish a model	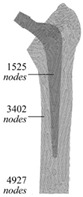	[32]
Machine learning techniques		Increase of diagnostic accuracy; not mature enough	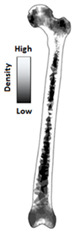	[33]

**Table 5:** A summary of the manufacturing techniques used for producing bone joint replacements.

Manufacturing Method		Representative Example	Advantages and Disadvantages	References
Traditional manufacturing techniques	Rapid prototyping (RP)	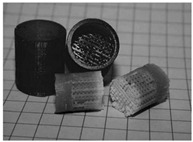	Advantages: Relative high precision; Disadvantages: The processing route is not easy to control; the cost is high	[35,37]
	Computer Numerical Control (CNC)	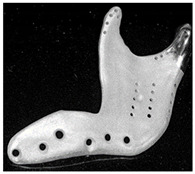		
Additive manufacturing techniques	Selective laser melting	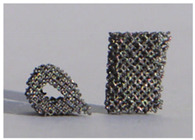	Advantages: Easy to Relative high precisionincorporate multiple materials, no support structure; Relative high precisionDisadvantages: Relatively poor mechanical properties	[39,40,41]
	Electron beam melting	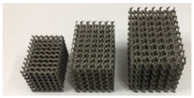		
	Selective laser sintering	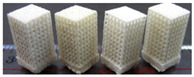		

**Table 6:** A summary of the in silico methods for evaluating the performance of knee joint replacements.

Type of Method	Advantages and Disadvantages	Representative Example	References
Musculoskeletal model	Able to simulate the activities of the human body; wear mechanism was not considered	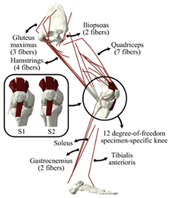	[44]
	Combines the advantages of numerical and experimental methods	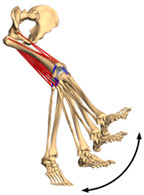	[45]
Coupled musculoskeletal-FE model	Reflects the stress state more comprehensively; lack of in vivo measurements	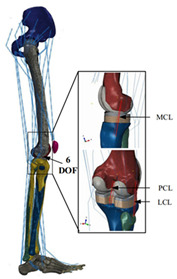	[46]
	Deformable contact models of the hip are considered; the accuracy can be improved	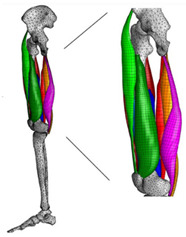	[47]
FE model	The mechanical information is comprehensive; difficult to develop the model	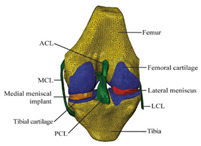	[48]
	The effect of simulation prediction is good	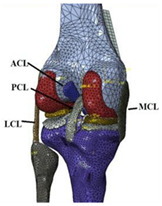	[49]

**Table 7:** The widely used in vitro testing methods for evaluating the performance of bone joint replacements.

Type of Method	Performance to Be Evaluated	Representative Study	References
Uniaxial tensile test	Strains on bones’ and prosthesis’ surfaces	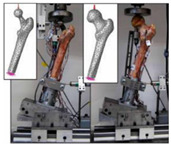	[51]
Static mechanical test	Stress shielding	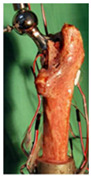	[53]
Laxity Test	The stress on the bone	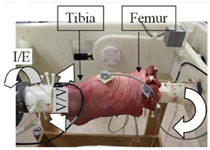	[49]
Fatigue test	The fatigue strength of the bone interface	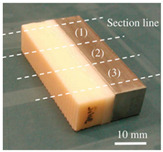	[52]

**Table 8:** The widely used in vivo testing methods for evaluating the performance of bone joint replacement.

Type of Method	Subjects to Be Used	Performance to Be Evaluated	Conclusion of Study	References
Animal testing	Goat metatarsus	Implant stability	The implant was identified as achieving favorable implant stability	[54]
	Tibia of rats	Prosthesis stress; implant stability	Implants with a pore size of 600 µm showed higher fixation ability than those with a pore size of 300 µm	[55]
	Tibia of rats	Release of osteopromotive molecules	Local controlled delivery of ZA alone can enhance bone implant anchorage	[58]
	Tibia of merino sheep	The rate of degradation; prosthesis stress	Iron-based porous materials can be candidates for the development of self-degrading bone replacement materials	[57]
	Knee of rats	The cytocompatibility	The multilayer scaffold could induce osteochondral repair	[59]
Clinical trial	Human temporomandibular joint	Prosthesis stress	The new implant has improved clinical and biomechanical joint function compared to the stock device	[56]
	Human mandibular	Bone formation	It is possible to form a mandibular replacement inside the latissimus dorsi muscle in a human being	[60]

## Data Availability

Not applicable.

## References

[1] Ferguson R.J., Palmer A.J., Taylor A., Porter M.L., Malchau H., Glyn-Jones S. (2018). Hip replacement. Lancet.

[2] Pivec R., Johnson A.J., Mears S.C., Mont M.A. (2012). Hip arthroplasty. Lancet.

[3] Matsoukas G., Willing R., Kim I.Y. (2009). Total hip wear assessment: A comparison between computational and in vitro wear assessment techniques using ISO 14242 loading and kinematics. J. Biomech. Eng..

[4] Barrack R.L. (2003). Dislocation after total hip arthroplasty: Implant design and orientation. Jaaos-J. Am. Acad. Orthop. Surg..

[5] Wang X., Xu S., Zhou S., Xu W., Leary M., Choong P., Xie Y. (2016). Topological design and additive manufacturing of porous metals for bone scaffolds and orthopaedic implants: A review. Biomaterials.

[6] Ścigała K., Będziński R., Filipiak J., Chlebus E., Dybała B. (2011). Application of generative technologies in the design of reduced stiffness stems of hip joint endoprosthesis. Arch. Civ. Mech. Eng..

[7] Shaik S.A., Bose K., Cherukuri H.P. (2012). A study of durability of hip implants. Mater. Des..

[8] Kladovasilakis N., Tsongas K., Tzetzis D. (2020). Finite element analysis of orthopedic hip implant with functionally graded bioinspired lattice structures. Biomimetics.

[9] Abdellah A.M., Fischer J., Yadav R., Khandaker M. (2017). Minimizing stress shielding and cement damage in cemented femoral component of a hip prosthesis through computational design optimization. Adv. Orthop..

[10] Nowak M., Sokołowski J., Żochowski A. (2020). New aspects of the trabecular bone remodeling regulatory model resulting from the shape optimization studies. Proc. Inst. Mech. Eng. Part H J. Eng. Med..

[11] Nowak M., Boguszewski A. (2021). Topology optimization without volume constraint–the new paradigm for lightweight design. Bull. Pol. Acad. Sci. Tech. Sci..

[12] Kokubo T., Yamaguchi S. (2016). Biomineralization of metals using chemical and heat treatments. Biominer. Biomater. Woodhead Publ..

[13] Guo Y., Liu K., Yu Z. (2018). Porous Structure Design in Tissue Engineering Using Anisotropic Radial Basis Functions. International Symposium on Visual Computing.

[14] Zheng Y.M. (2019). Bioinspired Design of Materials. Surf. Biol. Des. Mater..

[15] Al-Ketan O., Abu Al-Rub R.K. (2019). Multifunctional mechanical metamaterials based on triply periodic minimal surface lattices. Adv. Eng. Mater..

[16] Kolken H.M.A., de Jonge C.P., van der Sloten T., Garcia A.F., Pouran B., Willemsen K., Zadpoor A.A. (2021). Additively manufactured space-filling meta-implants. Acta Biomater..

[17] Bruno J., Brailovski V., Simoneau C., Dumas M., Terriault P. (2018). Development and in vitro validation of a simplified numerical model for the design of a biomimetic femoral stem. J. Mech. Behav. Biomed. Mater..

[18] Zhong Z. (2010). Progress in the study of mechanics problems of functionally graded materials and structures. Prog. Mech..

[19] Lin D., Li Q., Li W., Zhou S., Swain M.V. (2009). Design optimization of functionally graded dental implant for bone remodeling. Compos. Part B Eng..

[20] Singh G., Marwaha A. (2015). A review of metamaterials and its applications. IJETT.

[21] Liu Y., Hu H. (2010). A review on auxetic structures and polymeric materials. Sci. Res. Essays.

[22] Hou X., Silberschmidt V.V. (2015). Metamaterials with negative poisson’s ratio: A review of mechanical properties and deformation mechanisms. Mech. Adv. Mater..

[23] Kolken H.M.A., Garcia A.F., Du Plessis A., Rans C., Mirzaali M.J., Zadpoor A.A. (2021). Fatigue performance of auxetic meta-biomaterials. Acta Biomater..

[24] Yao Y., Wang L., Li J., Tian S., Zhang M., Fan Y. (2020). A novel auxetic structure based bone screw design: Tensile mechanical characterization and pullout fixation strength evaluation. Mater. Des..

[25] Bucher I., Ewins D.J. (2001). Modal analysis and testing of rotating structures. Philos. Trans. R. Soc. Lond. Ser. A Math. Phys. Eng. Sci..

[26] Gu Y. (2005). Meshfree methods and their comparisons. Int. J. Comput. Methods.

[27] Liu G.R., Zhang G.Y., Gu Y., Wang Y.Y. (2005). A meshfree radial point interpolation method (RPIM) for three-dimensional solids. Comput. Mech..

[28] Liu G.R. (2009). Meshfree Methods: Moving Beyond the Finite Element Method.

[29] Kononenko I. (2001). Machine learning for medical diagnosis: History, state of the art and perspective. Artif. Intell. Med..

[30] Cilla M., Checa S., Preininger B., Winkler T., Perka C., Duda G.N., Pumberger M. (2017). Femoral head necrosis: A finite element analysis of common and novel surgical techniques. Clin. Biomech..

[31] Hussin M.S., Fernandez J., Ramezani M., Kumar P., Kelly P.A. (2020). Analytical and computational sliding wear prediction in a novel knee implant: A case study. Comput. Methods Biomech. Biomed. Eng..

[32] Belinha J. (2016). Dinis, L.M.D.J.S.; Jorge, R.N. The analysis of the bone remodelling around femoral stems: A meshless approach. Math. Comput. Simul..

[33] Cilla M., Borgiani E., Martínez J., Duda G.N., Checa S. (2017). Machine learning techniques for the optimization of joint replacements: Application to a short-stem hip implant. PLoS ONE.

[34] Bhaskar B., Arun S., Sreekanth P.R., Kanagaraj S. (2016). Biomaterials in Total Hip Joint Replacements: The Evolution of Basic Concepts, Trends, and Current Limitations—A Review. Trends Biomater..

[35] Woesz A., Rumpler M., Stampfl J., Varga F., Fratzl-Zelman N., Roschger P., Fratzl P. (2005). Towards bone replacement materials from calcium phosphates via rapid prototyping and ceramic gelcasting. Mater. Sci. Eng. C.

[36] Ruyu M., Wendong X., Dongmei W., Kerong D., Chengtao W. (2005). Design and manufacture of custom hip prostheses based on standard X-ray films. Int. J. Adv. Manuf. Technol..

[37] Marcin K., Wach T., Szymor P., Zieliński R. (2017). Two different techniques of manufacturing TMJ replacements–a technical report. J. Cranio-Maxillofac. Surg..

[38] Bidyut P., Gupta S. (2020). The Relevance of Biomechanical Analysis in Joint Replacements: A Review. J. Inst. Eng. Ser. C.

[39] Wauthle R., Van Der Stok J., Yavari S A., Van Humbeeck J., Kruth J.P., Zadpoor A.A., Schrooten J. (2015). Additively manufactured porous tantalum implants. Acta Biomater..

[40] Ataee A., Li Y., Fraser D., Song G., Wen C. (2018). Anisotropic Ti-6Al-4V gyroid scaffolds manufactured by electron beam melting (EBM) for bone implant applications. Mater. Des..

[41] Duan B., Wang M., Zhou W., Cheung W.L., Li Z., Lu W. (2010). Three-dimensional nanocomposite scaffolds fabricated via selective laser sintering for bone tissue engineering. Acta Biomater..

[42] Arabnejad S., Johnston R.B., Pura J.A., Singh B., Tanzer M., Pasini D. (2016). High-strength porous biomaterials for bone replacement: A strategy to assess the interplay between cell morphology, mechanical properties, bone ingrowth and manufacturing constraints. Acta Biomater..

[43] Zhang K., Cheng G., Xu L. (2019). Topology optimization considering overhang constraint in additive manufacturing. Comput. Struct..

[44] Navacchia A., Hume D.R., Rullkoetter P.J., Shelburne K.B. (2019). A computationally efficient strategy to estimate muscle forces in a finite element musculoskeletal model of the lower limb. J. Biomech..

[45] Kebbach M., Grawe R., Geier A., Winter E., Bergschmidt P., Kluess D., Bader R. (2019). Effect of surgical parameters on the biomechanical behaviour of bicondylar total knee endoprostheses–A robot-assisted test method based on a musculoskeletal model. Sci. Rep..

[46] Shu L., Yamamoto K., Yao J., Saraswat P., Liu Y., Mitsuishi M., Sugita N. (2018). A subject-specific finite element musculoskeletal framework for mechanics analysis of a total knee replacement. J. Biomech..

[47] Li J., Lu Y., Miller S.C., Jin Z., Hua X. (2019). Development of a finite element musculoskeletal model with the ability to predict contractions of three-dimensional muscles. J. Biomech..

[48] Shriram D., Kumar G.P., Cui F., Lee Y.H.D., Subburaj K. (2017). Evaluating the effects of material properties of artificial meniscal implant in the human knee joint using finite element analysis. Sci. Rep..

[49] Beidokhti H.N., Janssen D., van de Groes S., Hazrati J., Van den Boogaard T., Verdonschot N. (2017). The influence of ligament modelling strategies on the predictive capability of finite element models of the human knee joint. J. Biomech..

[50] Matsoukas G., Kim I.Y. (2009). Design optimization of a total hip prosthesis for wear reduction. J. Biomech. Eng..

[51] Katz Y., Lubovsky O., Yosibash Z. (2018). Patient-specific finite element analysis of femurs with cemented hip implants. Clin. Biomech..

[52] Yang D., Zhang D., Arola D.D. (2010). Fatigue of the bone/cement interface and loosening of total joint replacements. Int. J. Fatigue.

[53] Zohar Y., Katz A., Milgrom C. (2013). Toward verified and validated FE simulations of a femur with a cemented hip prosthesis. Med Eng. Phys..

[54] Li G., Wang L., Pan W., Yang F., Jiang W., Wu X., Kong X., Dai K., Hao Y. (2016). In vitro and in vivo study of additive manufactured porous Ti6Al4V scaffolds for repairing bone defects. Sci. Rep..

[55] Taniguchi N., Fujibayashi S., Takemoto M., Sasaki K., Otsuki B., Nakamura T., Matsuda S. (2016). Effect of pore size on bone ingrowth into porous titanium implants fabricated by additive manufacturing: An in vivo experiment. Mater. Sci. Eng. C.

[56] Ackland D.C., Robinson D., Redhead M., Lee P.V.S., Moskaljuk A., Dimitroulis G. (2017). A personalized 3D-printed prosthetic joint replacement for the human temporomandibular joint: From implant design to implantation. J. Mech. Behav. Biomed. Mater..

[57] Wegener B., Sichler A., Milz S., Sprecher C., Pieper K., Hermanns W., Quadbeck P. (2020). Development of a novel biodegradable porous iron-based implant for bone replacement. Sci. Rep..

[58] Raina D.B., Larsson D., Sezgin E.A., Isaksson H., Tägil M., Lidgren L. (2019). Biomodulation of an implant for enhanced bone-implant anchorage. Acta Biomater..

[59] Du Y., Liu H., Yang Q., Wang S., Wang J., Ma J., Zhang S. (2017). Selective laser sintering scaffold with hierarchical architecture and gradient composition for osteochondral repair in rabbits. Biomaterials.

[60] Warnke P.H., Springer I.N.G., Wiltfang J., Acil Y., Eufinger H., Wehmöller M., Terheyden H. (2004). Growth and transplantation of a custom vascularised bone graft in a man. Lancet.

